# Preservation of Cognitive Function by *Lepidium meyenii* (Maca) Is Associated with Improvement of Mitochondrial Activity and Upregulation of Autophagy-Related Proteins in Middle-Aged Mouse Cortex

**DOI:** 10.1155/2016/4394261

**Published:** 2016-08-28

**Authors:** Shan-Shan Guo, Xiao-Fang Gao, Yan-Rong Gu, Zhong-Xiao Wan, A-Ming Lu, Zheng-Hong Qin, Li Luo

**Affiliations:** ^1^School of Physical Education and Sports Science, Soochow University, Suzhou 215021, China; ^2^Department of Nutrition and Food Hygiene, School of Public Health, Soochow University, 199 Renai Road, Suzhou 215123, China; ^3^Department of Pharmacology and Laboratory of Aging and Nervous Diseases, Jiangsu Key Laboratory of Preventive and Translational Medicine for Geriatric Diseases, Jiangsu Key Laboratory of Translational Research and Therapy for Neuro-Psycho-Diseases, Soochow University School of Pharmaceutical Science, Suzhou 215123, China

## Abstract

Maca has been used as a foodstuff and a traditional medicine in the Andean region for over 2,000 years. Recently the neuroprotective effects of maca also arouse interest of researchers. Decrease in mitochondrial function and decline in autophagy signaling may participate in the process of age-related cognitive decline. This study aimed to investigate if maca could improve cognitive function of middle-aged mice and if this effect was associated with improvement of mitochondrial activity and modulation of autophagy signaling in mouse cortex. Fourteen-month-old male ICR mice received maca powder administered by gavage for five weeks. Maca improved cognitive function, motor coordination, and endurance capacity in middle-aged mice, accompanied by increased mitochondrial respiratory function and upregulation of autophagy-related proteins in cortex. Our findings suggest that maca is a newly defined nutritional plant which can improve mitochondrial function and upregulate autophagy-related proteins and may be an effective functional food for slowing down age-related cognitive decline.

## 1. Introduction

*Lepidium meyenii* (maca) has been used as food and a traditional medicine in the Andean region for over 2,000 years [1]. Recently maca has been developed as dietary supplement for its potential advantageous effects on physical and sexual activity [2–4]. Multiple biological functions of maca have been demonstrated by human and animal studies, including enhancing sexual drive and fertility in men and women [2, 3], increasing vigor and energy levels [5], and reducing depression [6, 7].

Recently potential neuroprotective effects of maca have been studied in both* in vitro* and* in vivo* experimental models. A significant concentration-dependent protective effect of maca was observed in H_2_O_2_-treated crayfish neurons [8]. In another rat stroke model, the pentane extract of maca at lower dose (3 mg/kg per injection; 30 min prior to stroke; and 1 h after stroke) decreased infarct volumes, and higher doses (10 and 30 mg/kg per injection, resp.; 30 min prior to stroke and 1 h after stroke) increased infarct volumes compared to controls [8]. Previous studies have also shown that maca could improve learning and memory in some experimental animal models such as ethanol-, scopolamine-, or ovariectomy-induced memory impairment [9–11]. However, it is still not clear whether maca has some prophylactic effects on the age-related cognitive decline.

Mitochondria play a pivotal role in aging and are closely related to the earlier stages of some events that result in aging phenotype [12]. With age, changes in mitochondrial morphology and respiratory function could increase the production of reactive oxygen species (ROS) and thus affect cell homeostasis [12]. Mounting evidence indicated that age-dependent increase in mitochondrial dysfunction is involved in brain aging and neurodegenerative diseases [13]. Therefore, mitochondria are increasingly considered to be a target for preventing brain aging. Neurons are highly dependent on mitochondrial respiratory function due to their unique function and bioenergetic requirements [14]. Thus we proposed that maca can preserve cognitive function in middle-aged mice through improving mitochondrial function.

Autophagy, which is a cellular catabolic mechanism essential for degradation of misfolded proteins and dysfunctional organelles, has been implicated in brain aging and multiple neurodegenerative diseases [15]. Lipinski et al. demonstrated that autophagy was transcriptionally downregulated during aging in the human brain [15]. Pharmacological activation of autophagy has been shown to facilitate the clearance of intracellular protein aggregates such as *α*-synuclein [16] and amyloid *β* [17, 18] and promote neuronal survival in a series of disease models [19, 20]. It has been reported that diets containing 6% (w/w) walnuts can effectively activate autophagy in the striatum and hippocampus of 19-month-old rats [21] and improve cognitive function [22]. Thereby, we think it is of interest to investigate whether maca has some effects on autophagy signaling, consequently improving the cognitive function of middle-aged mice.

Existing evidence revealed that neuroprotective effects of maca may be related to improvement of antioxidant activity and reduction in oxidative stress [7, 9–11, 23]. Mitochondria are a main source of reactive oxygen species (ROS) and also a target of ROS at the same time. Autophagic turnover of cellular constituents is of great importance, especially in eliminating dysfunctional or damaged mitochondria, thus counteracting degeneration [24]. The purpose of this study was to investigate if the preservation of cognitive function by maca in middle-aged mice was associated with improvement of mitochondrial activity and modulation of autophagy signaling.

## 2. Materials and Methods

### 2.1. Plant Materials and Chemical Properties

**Figure 1:**
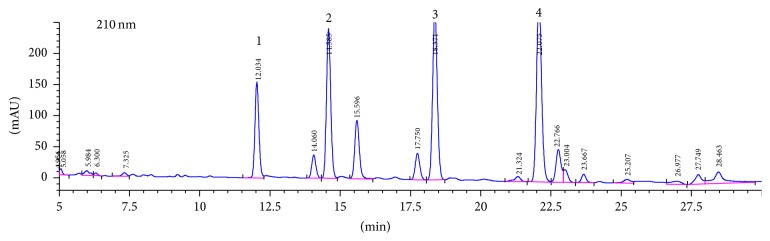
Analysis of macamides in maca powder measured by HPLC using standard maca product from Peru as a reference. (1–4): macamides.

Maca powder was imported from Peru and was a gift from Nanjing Bio-Array Technology Company (Nanjing, China) and was chemically identified by Suzhou Institute of Chinese Materia Medica, Suzhou University. The macamides have been considered to be the signature compounds of maca [25] and have shown promising pharmacological activities in some studies [26, 27]. Thus the macamides were measured by high-performance liquid chromatography (HPLC) using standard maca product from Peru as a reference. The results proved that maca powder used in this study has similar levels of active compounds as the standard (Figure 1).

The chemical properties of maca powder used were shown in Table 1.

### 2.2. Animals and Treatment

**Figure 2:**
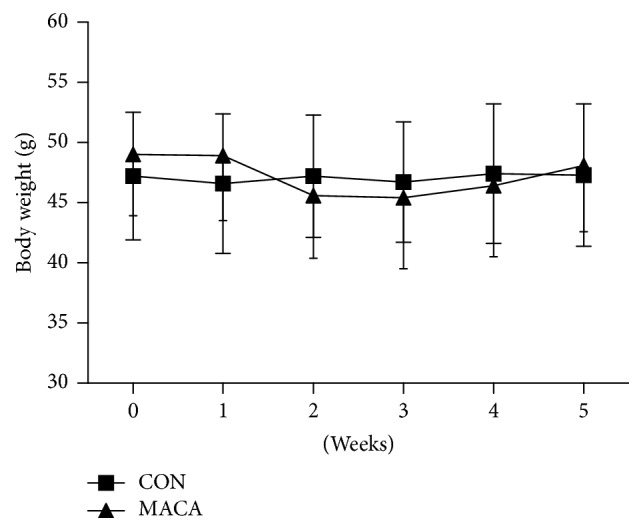
The effects of maca on body weight of middle-aged mice.

**Figure 3:**
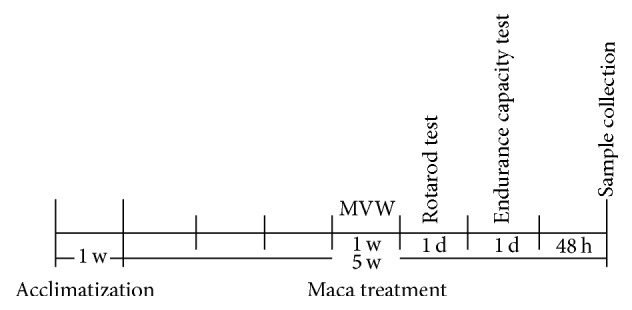
Timeline for experimental design. The mice were allowed to acclimatize to the environment for the first week. Then the mice in MACA were treated with maca powder suspension administered by gavage once a day for 5 weeks. From the 25th day of maca treatment, all mice underwent Morris water maze test (1 w), rotarod test (1 d), and endurance capacity test (1 d) in succession. Sample collection was performed 48 h after the endurance capacity test to minimize the influence of behavioral tests.

Fourteen middle-aged (14-month-old) male ICR mice (46.1 ± 5.1 g) were obtained from the Experimental Animal Center of Soochow University. One week after arriving at the facility, mice were randomly assigned to two groups: (1) control group (CON, *n* = 7), (2) maca-treated group (MACA, *n* = 7). Maca powder (150 meshes) was suspended in saline at 50 mg/mL. The mice in MACA received maca powder suspension (0.1 mL/10 g body weight, 500 mg maca powder/kg body weight) administered by gavage once a day for 5 weeks. The mice in CON received equal volumes of saline with the same method. All mice were kept in individual cages with standard food and water* ad libitum* in a temperature (22°  ±  2.5°C) and light-controlled (12 : 12 h light-dark cycle) environment. The body weights of mice were recorded every week (Figure 2). The study protocols were approved by Animal Care Ethical Committee of Soochow University. The experiment design was shown in Figure 3.

### 2.3. Behavioral Tests

#### 2.3.1. Morris Water Maze Test

Morris water maze (MWM) test was similar to those described in a previous study [28]. Briefly, all mice were tested for spatial learning and memory performance with the Morris water maze. The maze consisted of a 1.2 m diameter circular white fiberglass pool filled to a depth of 50 cm with water (26 ± 1°C) made opaque with the addition of nontoxic white latex paint and was in a room with extra maze cues on the walls around the pool. A circular escape platform (10 cm diameter) was submerged approximately 1 cm below the water surface in one of the quadrants of the pool, and this position remained constant throughout testing. All mice were first habituated to the maze with a 60 s free-swim in the pool prior to testing. Mice then completed 24 trials over 6 consecutive days to learn the location of the submerged platform (the spatial acquisition phase, four trials per day; 60 s maximum trial duration). If the mice did not find the platform within 60 seconds, it was placed on the platform for 15 seconds. Latencies to locate the hidden platform were monitored by a video camera mounted in the ceiling and a computerized tracking system (ANY-maze video tracking system, Stoelting Co.). On day 7, the mice were given a 60 s retention test of the spatial location (probe test) with the platform absent, and the times of crossing the previous hidden platform were recorded.

#### 2.3.2. Rotarod Assessment of Motor Coordination

Then motor coordination was assessed with a rotarod treadmill (San Diego Instruments, San Diego, CA, USA) according to a previous study [29] with minor modifications. Mice were allowed to acclimate to the rod at fixed speed (5 rpm/min) for 30 s. Then the mice were tested using the accelerated version of the rotarod test, in which the rotating speed was accelerated from 5 rpm/min to 50 rpm/min within 5 min. The fall-off latency was averaged from three tests.

#### 2.3.3. Measurement of Swimming Endurance Capacity

Swimming endurance capacity was assessed according to a previous study [30] with minor modifications. All mice were subjected to a progressive load test in the swimming apparatus (60 cm × 55 cm × 80 cm) in order to determine endurance capacity. The mice were placed in the water (26 ± 1°C, 45 cm in depth) with a load (lead wire) attached to the tail corresponding to 1% of their body weight, with a step increase in weight (1% of each mouse's body weight) every 3 min until exhaustion (determined by 10 continuous seconds submerged). The time to exhaustion was recorded as the endurance capacity of each mouse.

### 2.4. Tissue Harvesting

Forty-eight h after endurance capacity test, the mice were sacrificed by decapitation under anesthesia and the cortex were dissected and stored at −80°C prior to analysis.

### 2.5. Immunoblotting

Frozen cortical tissues were lysed and used for immunoblotting as described previously [31]. Equal amounts (30–45 *μ*g) of total protein extracts were separated by 10–15% SDS-PAGE and the separated proteins were transferred onto nitrocellulose membranes. Nonspecific binding was blocked by incubating membranes in Tris buffered saline containing 0.05% Tween 20 (v/v) and 5% nonfat milk (v/v) for 1 h. Blots were incubated with primary antibodies as follows: LC3 A/B (1 : 1000; Abcam), Atg7 (1 : 1000; Beyotime Institute of Biotechnology), Beclin1 (1 : 1000, Cell Signaling), Total OXPHOS Rodent WB Antibody Cocktail (1 : 250, Abcam), and *β*-actin (1 : 5000; Sigma) at 4°C overnight. The membranes were washed and incubated with IRDye secondary antibodies (1 : 10,000; Li-Cor Bioscience) for 1 h at room temperature. The images of protein-antibody interaction were captured with the Odyssey infrared imaging system (Li-Cor Bioscience) and analyzed with Image J with normalization to the loading control *β*-actin.

### 2.6. Statistical Analysis

For the MWM test, escape latencies were analyzed with repeated measures analysis of variance (ANOVA). One-way ANOVA and Newman-Keuls post hoc tests (two-tailed) were performed to determine the differences of escape latencies between groups at different time point and the differences of times of crossing the previous hidden platform between groups in the probe test. For the Western blot analysis and rotarod test, nonparametric Mann-Whitney *U* test (two-tailed) was used. Differences were considered significant when *p* < 0.05.

## 3. Results

### 3.1. Maca Improved Spatial Learning and Memory in Middle-Aged Mice

**Figure 4:**
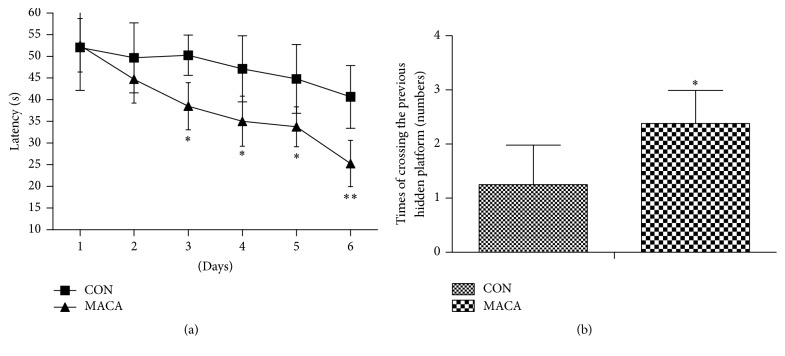
Maca improves spatial learning and memory of middle-aged mice. Values are the mean ± SD. ^*∗*^ *p* < 0.05 and ^*∗∗*^ *p* < 0.01 versus CON.

Learning and memory capacity was assessed in all mice using the Morris water maze test. Mice were tested in the hidden platform version of the water maze for 6 consecutive days, and goal latencies were evaluated. During the spatial acquisition phase, mice in MACA spent less time in locating the hidden platform than CON mice on days 3, 4, 5, and 6, respectively (Figure 4). A probe trial test was performed on day 7. Mice in maca-treated group showed an increase of times of crossing the previous hidden platform compared with mice in CON, suggesting an improvement in memory retention (Figure 4).

### 3.2. Maca Improved Motor Coordination and Swimming Endurance Capacity in Middle-Aged Mice

**Figure 5:**
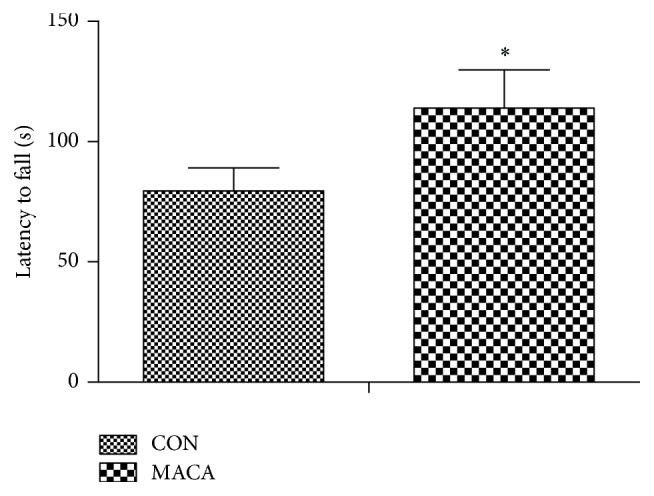
Maca improves motor coordination of middle-aged mice. Values are the mean ± SD. ^*∗*^ *p* < 0.05 versus CON.

Motor coordination was assessed after five weeks of maca treatment with a rotarod treadmill. An increase in latency to fall in maca-treated mice was shown in Figure 5. This finding suggests an improved motor coordination ability of mice after maca treatment.

**Figure 6:**
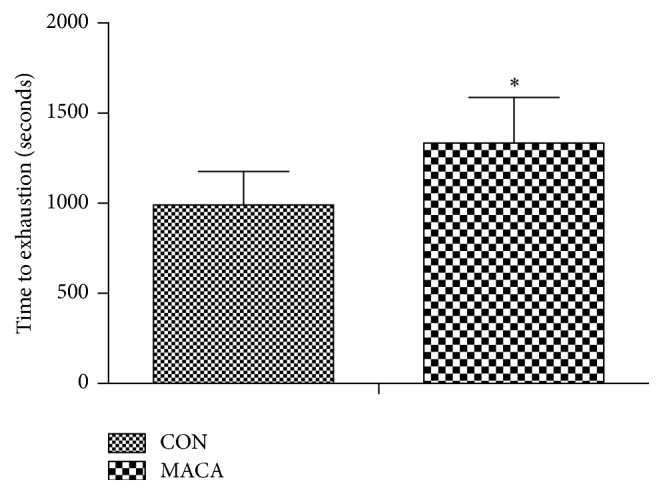
Maca improves endurance capacity in middle-aged mice. All mice were subject to a progressive load test in the swimming apparatus to determine the endurance capacity. Values are the mean ± SD. ^*∗*^ *p* < 0.05 versus CON.

The swimming time to exhaustion was measured to investigate if maca had the antifatigue property in middle-aged mice. As shown in Figure 6, the swimming time of the mice in MACA was significantly higher than that in CON.

### 3.3. Maca Increased the Expression of Subunits of Mitochondrial Respiratory Chain Complex in the Cortex of Middle-Aged Mice

**Figure 7:**
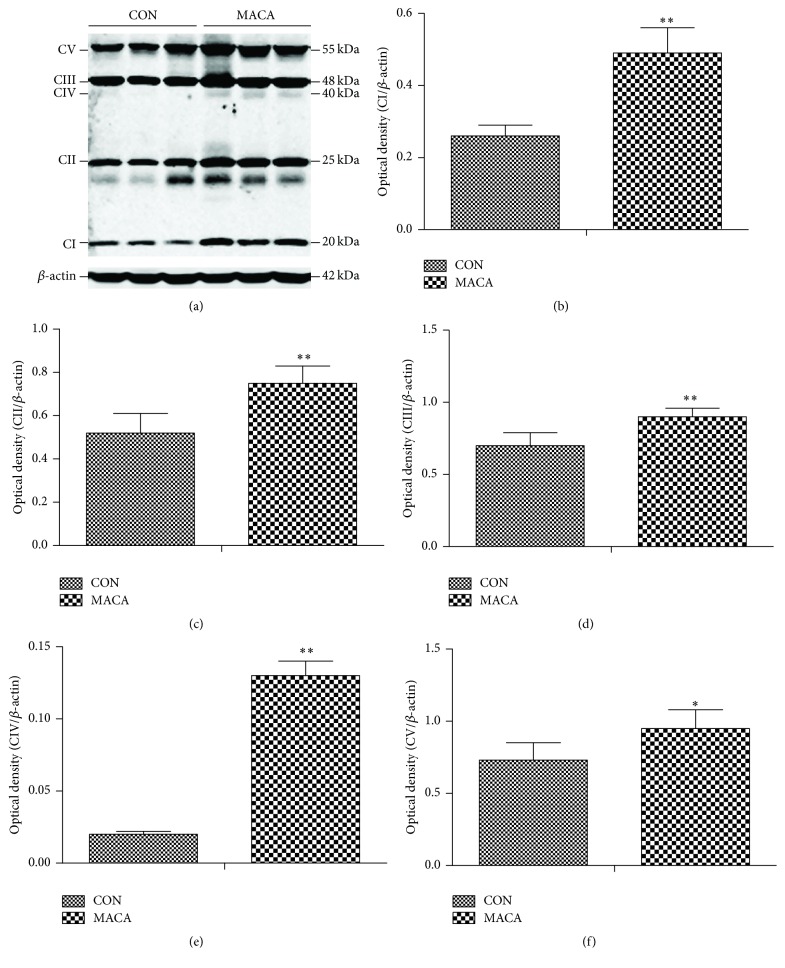
Maca increases the protein expression of subunits of mitochondrial respiratory chain complexes in cortex of middle-aged mice. The cortex was extracted from different groups of mice and subjected to Western blot analysis of OXPHOS. Representative immunoblot images (a) and quantification of mitochondrial OXPHOS complexes I–IV (b–f) in the hippocampal extracts of mice. Values are the mean ± SD. ^*∗*^ *p* < 0.05 and ^*∗∗*^ *p* < 0.01 versus CON.

The protein levels of oxidative phosphorylation (OXPHOS) enzyme complexes have been used as indicator of mitochondrial metabolic function in previous studies [32–37]. As shown in Figure 7, there were significant increases in OXPHOS I, II, III, IV, and V complexes in the cortex of maca-treated middle-aged mice, when compared to the age-matched controls, suggesting an improvement of mitochondrial respiratory function in the cortex of maca-treated mice.

### 3.4. Maca Upregulated the Expression of Autophagy-Related Proteins in Cortex of Middle-Aged Mice

**Figure 8:**
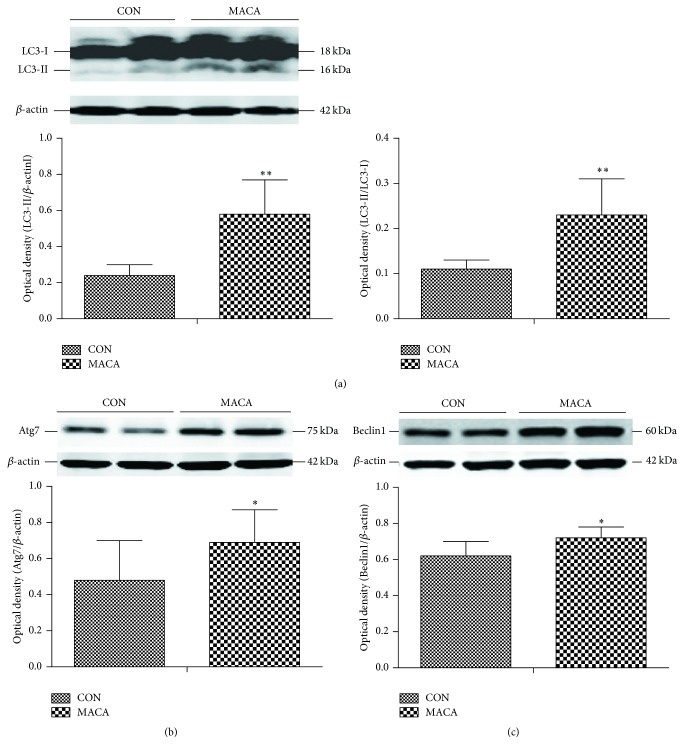
Maca upregulates autophagy-related proteins in cortex of middle-aged mice. The cortex was extracted from different groups of mice and subjected to Western blot analysis of LC3, Atg7, and Beclin1. (a) LC3; (b) Atg7; (c) Beclin1. Values are the mean ± SD. ^*∗*^ *p* < 0.05 and ^*∗∗*^ *p* < 0.01 versus CON.

Next we measured levels of LC3, Atg7, and Beclin1 to determine the effects of maca on autophagy-related proteins in cortex. The protein level of LC3-II and the ratio LC3-II/LC3-I were significantly higher in MACA than those in CON (Figure 8(a)). In addition, Atg7 and Beclin1 protein levels were also significantly higher in MACA than those in CON (Figures 8(b) and 8(c)). These data suggest that maca might activate autophagy signaling in cortex of middle-aged mice.

## 4. Discussion

In the present study, we demonstrated that five weeks of maca supplementation improved cognitive function in middle-aged mice. Besides, maca increased the protein levels of subunits of OXPHOS complexes and autophagy-related proteins in mouse cortex. These data suggest the improvement of cognitive function by maca may be associated with, at least partially, improvement of mitochondrial respiratory function and upregulation of autophagy-related proteins in cortex of middle-aged mouse.

Maca has been demonstrated to possess multiple biological properties, such as antifatigue, improving sexual performance and neuroprotective activities [3, 8, 27, 38]. In this study, supplementation of maca for five weeks significantly improved the endurance capacity and motor coordination in middle-aged mice, which was in accordance with previous studies [3, 38, 39]. Rubio et al. reported that aqueous and hydroalcoholic extracts of black maca improved memory deficits in mice induced by ethanol, scopolamine, or ovariectomy, respectively [9–11]. However, it is still not clear whether maca can improve learning and memory capacity in the middle-aged mice. In this study, the mice in MACA showed better learning and memory ability as assessed by the Morris water maze test, suggesting the potential of maca for preventing cognitive decline in the elderly.

Alterations of mitochondrial functions are linked to brain aging and a few neurodegenerative diseases. With age, mitochondria become progressively inefficient and generate more ROS, which will damage the macromolecules such as lipids, proteins, and carbohydrates [12]. Besides, damaged mitochondria can potentially trigger apoptosis and necrosis and thus lead to cell death [24]. Previous studies have demonstrated that some of the biological actions of maca, including improving endurance capacity and antifatigue property, were associated with the improvement of energy metabolism and antioxidant status [38]. In addition, aqueous extract of black maca has been reported to improve experimental memory impairment induced by ovariectomy via downregulation of oxidative stress [11]. Our results demonstrated for the first time that the neuroprotective effects of maca were accompanied by an improvement of mitochondrial respiratory function. Nevertheless, further studies are required to clarify the cause and effect relationship between reduction of oxidative stress and improvement of mitochondrial function by maca.

Dysregulation of autophagy has been considered to be involved in brain aging and multiple neurodegenerative diseases [15]. Moreover, autophagy/mitophagy plays a pivotal role in mitochondrial quality control by eliminating damaged or dysfunctional mitochondria [40]. Our present study showed that maca increased the protein level of LC3-II and the ratio of LC3-II/LC3-I, along with upregulation of Atg 7 and Beclin1 proteins, indicating autophagy signaling might be activated in the cortex of maca-treated middle-aged mice. Thus, the restoration of cognitive function in middle-aged mice by maca might also be associated with upregulation of autophagy-related proteins.

In summary, the present study demonstrated for the first time that maca improves cognitive function in middle-aged mice, and this effect may be associated with improved mitochondrial respiratory function and upregulation of autophagy-related proteins.

## Figures and Tables

**Table 1:** Chemical composition of maca powder.

Number	Specification	Unit per 100 g of product	Maca powder
1	Energy value	kJ	1313
2	Carbohydrates	g	46.1
3	Crude protein	g	21.9
4	Fat	g	0.9
5	Dietary fiber	g	15.6
